# Optical coherence tomography analysis of anterior segment parameters before and after laser peripheral iridotomy in primary angle-closure suspects by using CASIA2

**DOI:** 10.1186/s12886-022-02366-2

**Published:** 2022-03-28

**Authors:** Xiaoxiao Chen, Xiaolei Wang, Yizhen Tang, Xinghuai Sun, Yuhong Chen

**Affiliations:** 1 Department of Ophthalmology and Visual Science, Eye, Ear, Nose and Throat Hospital, Shanghai Medical College of Fudan University, Shanghai, 200031 China; 2 NHC Key Laboratory of Myopia, Chinese Academy of Medical Sciences, and Shanghai Key Laboratory of Visual Impairment and Restoration (Fudan University), , Shanghai, 200031 China; 3 State Key Laboratory of Medical Neurobiology and MOE Frontiers Center for Brain Science Institutes of Brain Science, Fudan University, Shanghai, 200032 China

**Keywords:** Anterior segment biometric parameters, Laser peripheral iridotomy, Anterior segment optical coherence tomography, Primary angle-closure suspects, CASIA 2

## Abstract

**Background:**

Laser peripheral iridotomy (LPI) is effective in primary angle-closure suspects (PACS); however, predictors for anterior segment alterations after LPI are limited. We aimed to evaluate the anterior segment biometric parameters before and after LPI in PACS using the recently developed, CASIA 2 device of anterior segment optical coherence tomography (AS-OCT).

**Methods:**

We performed LPI in 52 PACS. Anterior segment parameters, including anterior chamber depth (ACD), anterior chamber width (ACW), anterior chamber volume (ACV), iris curvature (ICURVE), iridotrabecular contact (ITC), lens vault (LV), lens thickness (LT), radius of the lens, angle opening distance (AOD), angle recess area (ARA), trabecular iris space area (TISA), and trabecular iris angle (TIA) at different distances (i.e., 500 μm from the sclera spur), were evaluated before and after LPI using CASIA 2.

**Results:**

Eyes of PACS after LPI had a greater ACV, AOD, ARA, TISA, and TIA, and a lower ITC and ICURVE (all *p* < 0.001) than those before LPI. On a 360° scan, the anterior chamber angle in the superior quadrant increased the most after the LPI. A higher baseline LT was significantly associated with a greater postoperative increase in AOD 500, ARA 500, TISA 500, and TIA 500 (*p* = 0.001, *p* = 0.010, *p* = 0.004, and *p* < 0.001, respectively).

**Conclusions:**

We found that LPI widens the anterior chamber angle in the PACS, especially, in the superior quadrant around the iridotomy hole. Eyes with a thicker lens are more likely to experience angle opening because of the LPI.

## Background

Glaucoma is the second leading cause of irreversible blindness, worldwide [1]. The two major types of glaucoma are primary open-angle glaucoma (POAG) and primary angle-closure glaucoma (PACG). Contrary to POAG, angle-closure is the fundamental pathogenic change in PACG, which leads to an elevation in the intraocular pressure (IOP) [2, 3]. Thus, the principle aim of PACG management is to keep the anterior chamber angle open and maintain a stable drainage function. Current treatment for PACG includes medications, laser therapy, and surgery. Laser peripheral iridotomy (LPI) is a safe and effective treatment that could help relieve the pupillary block and, thereby, reverse the appositional angle-closure, widen the angle, and prevent optic nerve damage caused by elevated IOP [4]. LPI has commonly been used as a prophylactic treatment to prevent disease progression in primary angle-closure suspects (PACS) [5]. However, LPI does not have a consistent effect in PACS; acute episodes can occur in a small proportion of patients despite LPI [6]. Therefore, analysis of the anterior segment structures before and after LPI would aid in understanding the underlying mechanism of LPI and identifying the right candidates for LPI.

With the increasing use of innovative anterior segment assessment techniques, such as ultrasound biomicroscopy (UBM) and anterior segment optical coherence tomography (AS-OCT), gradually precise measurement of many anterior segment parameters is possible. Compared to UBM, AS-OCT is easier to use and does not require any contact with the eye. The CASIA 2 (Tomey, Nagoya, Japan), a newly developed AS-OCT device with a swept-source laser wavelength of 1310 nm, has been demonstrated to have improved measuring accuracy and functionality. Anterior chamber volume (ACV) was previously e estimated by 360° rotation of several cross-sectional scans of the anterior chamber area; however, this technique was not accurate owing to the heterogeneity from different degrees. Moreover, scleral spur, which was previously used as the initial reference landmark for the sequential measures, can be difficult to identify on OCT [6]. However, CASIA 2 allows direct automated measurement of 360° ACV, iridotrabecular contact (ITC), lens parameters, and recognition of the scleral spur [7].

The finding presumed LPI in patients but rarely focused on lens parameters due to the incapability in measuring the posterior surface of the lens. ACV was also measured inaccurately owing to prediction according to some of the slices. The present study aimed to determine the changes in the anterior chamber and the lens parameters after LPI, observed using CASIA 2, and their influencing factors. In the technique, we adapted strategies to mitigate the impact of artificial errors caused by location of the scleral spur. Moreover, we could analyze the circumferential angle opening depending on CASIA 2. Based on the accurate measurement of lens parameters, we focused on the relationship between baseline lens parameters and angle opening degree to determine the predictors of target LPI patients.

## Methods

### Study participants

This was a prospective observational study. Participants were recruited between December 1, 2018, to December 23, 2018, from the glaucoma clinic of the Eye, Ear, Nose and Throat (EENT) Hospital of Fudan University of Shanghai, China. The study received approval from the Ethical Review Committee of EENT Hospital and adhered to the Declaration of Helsinki. Signed informed consent was obtained from all the participants before conducting any examination or operation.

Patients underwent a complete ophthalmologic examination, including slit-lamp biomicroscopy, visual acuity test, IOP measurement with Goldmann applanation tonometry, gonioscopy, axial length (AL), and central corneal thickness (CCT) measurement with ultrasound pachymetry (A-scan Pachymeter, Ultrasonic, Exton, PA, USA), frequency-domain optical coherence tomography (FD-OCT) (RTvue OCT; Optovue Inc., Toledo, OH), and standard automated perimetry (30–2 Swedish Interactive Threshold Algorithm; Humphery Field Analyzer II; Cal Zeiss Meditec, Inc., Dublin, CA). Demographic information, including age, sex, and laterality, were recorded.

The PACS were diagnosed based on the criteria developed in 2002 by the International Society of Geographic and Epidemiologic Ophthalmology and preferred practice pattern of American Academy of Ophthalmology [8, 9]. The inclusion criteria were as follows: (1) an eye with pigmented trabecular meshwork not visible for ≥ 180° on static gonioscopy and without peripheral anterior synechiae, (2) IOP ≤ 21 mm Hg, and (3) no optic nerve damage. Exclusion criteria were plateau iris, previous intraocular surgery, non-glaucomatous optic neuropathy, secondary elevated IOP, or other ocular diseases.

### Laser peripheral iridotomy

All LPIs were performed by the same ophthalmologist. Topical oxybuprocaine of 0.04 ml was applied to the eye for anesthesia. Neodymium-doped yttrium aluminum garnet (Nd: YAG) laser and an iridectomy lens (Volk Optical Inc, Mentor, Ohio, USA) were used to perform the iridotomy. All LPIs were performed approximately 1 mm from the limbus in the superior quadrant between the 10 and 2 o’clock positions (Fig. 2B, and Figure C). All patients were prescribed 1% prednisolone eye drops four times for the first hour, and four times a day for a week thereafter. Patients with an elevated IOP received topical ocular hypotensive eyedrops.

### Anterior segment optical coherence tomography

The postoperative scans using CASIA 2 were performed one week after LPI by the same operator at the same time of the day using the same scanning protocol. The anterior chamber angle and lens scan protocols of CASIA 2 were used to obtain a volume scan with a length and depth of 16 mm and 13 mm, respectively. Images were acquired during the 5 s while the patient was fixing on an internal target. Three-dimensional analysis was performed on 16 AS-OCT images from 16 different angles. Images with motion or lid artifacts were excluded.

The anterior chamber parameters included anterior chamber depth (ACD, the axial distance from the corneal endothelium to the anterior lens surface), anterior chamber width (ACW, the distance between the two scleral spurs), and ACV (the volume bordered by the posterior surface of the cornea and the anterior surface of the iris and lens). Iris indices, including iris volume, iris thickness at 750 μm from the scleral spur (IT 750), iris thickness at 2000 μm from the scleral spur (IT 2000), and iris curvature, were obtained. Iris curvature was the maximum distance between the posterior surface of the iris and the straight line connecting the iris root and the contact end point of the iris on the crystalline lens. The ITC before and after LPI were compared using the parameters of ITC index and ITC area. In CASIA 2, the ITC index was defined as the ratio of angle-closure in degrees to the total angle with visible scleral spur and end point in degrees [10]. The ITC area was defined as the area of the extent of the circumferential contact of peripheral iris to the angle wall [11]. All images were processed using inbuilt semi-automated software by a single experienced observer who was masked to clinical data (X.C). The scleral spurs were determined by two glaucoma specialists (X.W. & Y.C.).

**Fig. 1:**
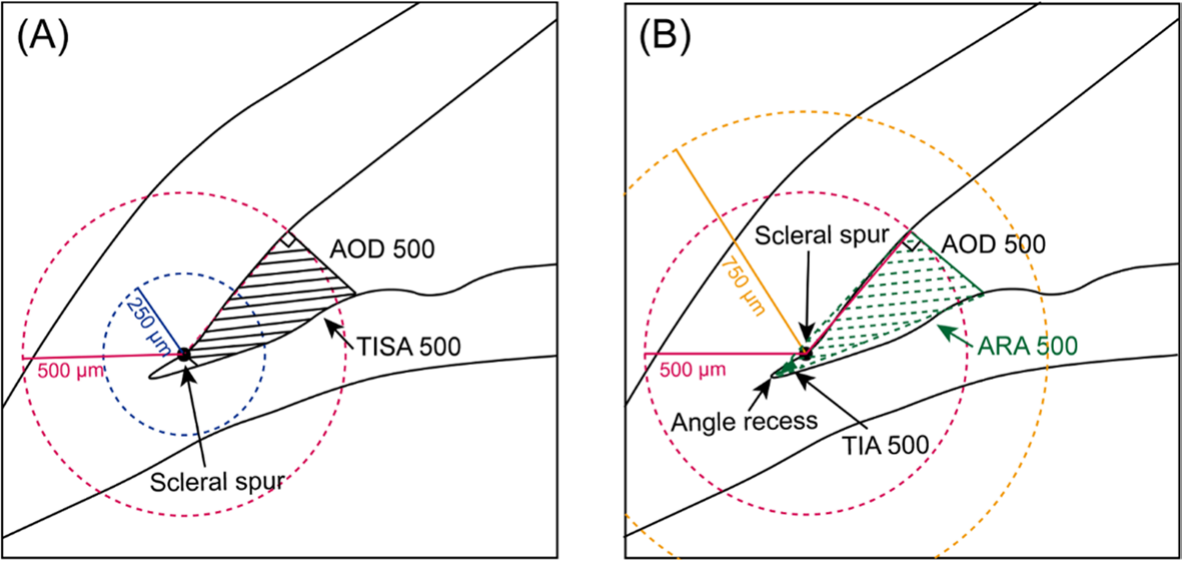
Quantitative determination of the anterior chamber angle parameters assessed by analysis software of CASIA 2. **A** Circles are drawn with scleral spur (long arrow) as center point and 250 μm (blue circle) and 500 μm (red circle). AOD 500 is defined as the distance perpendicular to the inner corneoscleral wall starting from the intersection point of the red circle and inner corneoscleral wall. TISA 500 (shaded area) is the trapezoidal area bounded by AOD 500, anterior iris surface, inner corneoscleral wall, and perpendicular distance from the scleral spur to the iris surface. **B** The circle is drawn with scleral spur (long arrow) as center point with 750 μm (orange circle) as radius. ARA 500 (green shaded area) is defined as the triangular area bounded by AOD 500 (green full line), inner corneoscleral wall, and iris surface. TIA 500 is the value of the apex angle with AOD 500 as the base of the triangle. AOD = angle opening distance, TISA = trabecular iris space area, ARA = angle recess area, TIA = trabecular iris angle

The parameters applied in angle analysis were angle opening distance (AOD), angle recess area (ARA), trabecular iris space area (TISA), and trabecular iris angle (TIA) [12]. AOD 250, AOD 500, and AOD 750 were defined as the distance between the posterior corneoscleral surface and the anterior iris surface on a line perpendicular to the trabecular meshwork at 250 μm, 500 μm, and 750 μm from the scleral spur, respectively (Fig. 1A). The ARA was defined as the triangular area formed by the AOD, the iris surface, and the inner corneoscleral wall intersected at the angle recess (Fig. 1B). The TIA was the value of the apex angle of this triangle at the angle recess (Fig. 1B). The TIA refers to a trapezoidal area formed by the following: AOD, a line parallel to AOD starting from the scleral spur and ending at the opposing iris, the inner corneoscleral wall, and the iris surface (Fig. 1A).

Automated circumferential (360°) angle parameters of AOD 500, ARA 500, TISA 500, and TIA 500 were measured and compared in different sectors. Anticlockwise rotation from 0º to 360º (16 section images: 0–180º, 11–191º, 23–203º, 34–214º, 45–225º, 56–236º, 68–248º, 79–259º, 90–270º, 101–281º, 113–293º,124–304º, 135–315º, 146–326º, 158–338º, 349–169º) matched nasal, superior, temporal, and inferior quadrants in the right eyes (Fig. 2A). The degrees of the left eyes were mirror transformed to have the same orientation as the right eyes. The percentage change of angle segment parameters was calculated by dividing the pre-LPI parameters by the difference between pre- and post-LPI (for e.g., mean AOD 500 differential rate was calculated as [(post-LPI mean AOD 500 at 1 week − baseline mean AOD 500)/baseline mean AOD 500] %, hereafter denoted as ΔAOD 500).

**Fig. 2:**
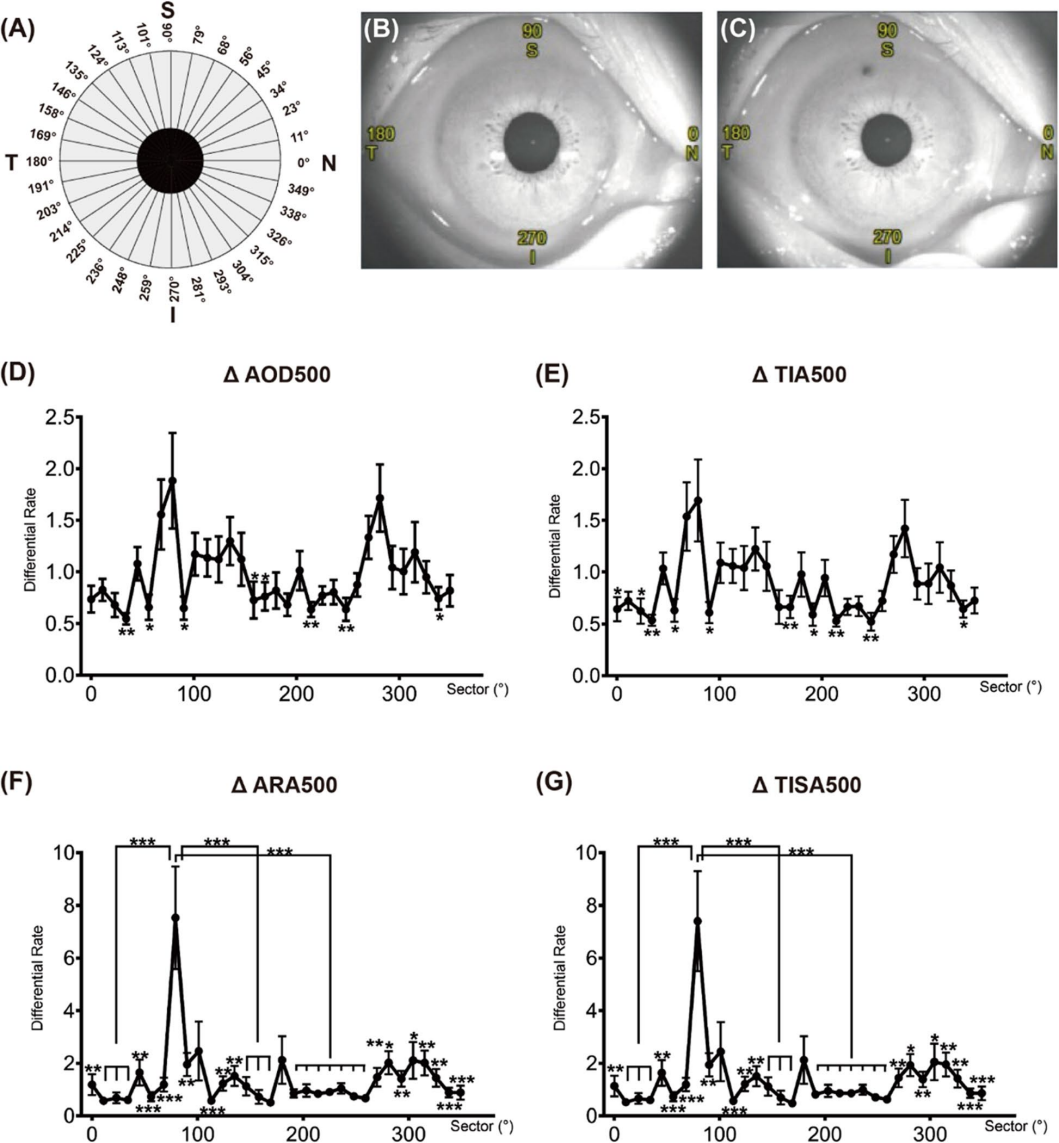
The change in anterior chamber parameters at different circumferential angles. Schematic representation of the right eye **A** divided by sixteen 2D sections with interval of approximately 11°. Preoperative **B** and postoperative **C** scans of the right eye of a primary angle-closure suspect shows the location of the laser peripheral iridotomy hole and the orientation degrees of 0°, 90°, 180°, and 270° representing nasal, superior, temporal, and inferior quadrants, respectively. **D** AOD 500 (angle opening distance at 500 μm from the scleral spur), **E** TIA 500 (trabecular iris angle at 500 μm from the scleral spur), **F** ARA 500 (angle recess area at 500 μm from the scleral spur), and **G** TISA 500 (trabecular iris space area at 500 μm from the scleral spur), are shown as differential ratios (Δ index = [postoperative index − preoperative index] / preoperative index). Repeated measurement data analysis of variance demonstrated that the ΔAOD 500, ΔTIA 500, ΔARA 500, and ΔTISA 500 were significantly different across sectors (F11.882, 13.985 = 6.652, F12.205, 11.643 = 6.887, F4.521, 539.625 = 14.928, F4.579, 517.686 = 14.962, respectively, all *p* < 0.001). Pairwise comparisons among sectors were conducted by post hoc analysis. The stars labeled on the line graph represents the p-values of the post hoc analysis comparing certain sector degrees to sector 79° (*, *p* < 0.05; **, *p* < 0.01; ***, *p* < 0.001)

Lens parameters, including the radii of curvature of anterior lens (front R) and posterior lens (back R), decentration, and tilt were analyzed [13, 14]. Additionally, the lens vault (LV) and lens thickness (LT) were recorded and compared.

### Statistical analysis

The Statistical Package for Social Sciences Version 23.0 for Mac (SPSS Inc., Chicago, IL) was applied for statistical analysis. *p*-value less than 0.05 was considered statistically significant. The categorical variables were described as percentages. All the continuous variables were presented as mean ± standard deviation. Differences between preoperative and postoperative measurements of the anterior chamber and lens parameters were compared by the paired Student's t-test. Repeated measurement data analysis of variance was applied to study the anterior chamber angle parameters of ΔAOD 500, ΔTIA 500, ΔTISA 500, and ΔARA 500 in different sectors. The Bonferroni test was used for pairwise comparisons. The multivariate linear regression model was used to analyze the predictors associated with the change in the anterior segment biometric parameters. The demographic variables (age and sex) and anterior segment variables listed in Table 1 except for AOD, ARA, TISA and TIA were initially included in the multivariate linear regression model. Variables that showed multicollinearity identified by correlation coefficient more than 0.7 were excluded. Both R^2^ and VIF (variance inflation factor) were considered simultaneously to develop the final multivariate regression model. Age, sex, ACD, IT 750, and LT were included in the final equation.

**Table 1:** Anterior segment parameters in primary angle-closure suspects before and after laser peripheral iridotomy

	**Preoperative (mean ± SD)**	**Postoperative (mean ± SD)**	***p-*value**^**†**^
AC indexes (mm)			
ACD	2.03 ± 2.27	2.04 ± 0.28	0.151
ACW	11.38 ± 0.40	11.38 ± 0.38	0.352
ACV	79.80 ± 4.26	92.29 ± 19.48	< 0.001
Iris Indexes			
Iris Volume (mm^2^)	34.38 ± 4.26	34.22 ± 3.94	0.428
IT 750 (mm)	0.33 ± 0.06	0.32 ± 0.05	0.006
IT 2000 (mm)	0.40 ± 0.06	0.39 ± 0.06	0.044
Iris Curvature (mm)	0.27 ± 0.06	0.09 ± 0.07	< 0.001
ITC Index (%)	26.43 ± 3.67	18.64 ± 2.60	< 0.001
ITC Area (mm^2^)	6.35 ± 0.71	1.75 ± 0.38	< 0.001
AOD (mm)			
AOD 250	0.08 ± 0.04	0.15 ± 0.06	< 0.001
AOD 500	0.10 ± 0.56	0.21 ± 0.09	< 0.001
AOD 750	0.14 ± 0.08	0.29 ± 0.13	< 0.001
ARA (mm^2^)			
ARA 250	0.02 ± 0.01	0.04 ± 0.02	< 0.001
ARA 500	0.04 ± 0.02	0.08 ± 0.03	< 0.001
ARA 750	0.07 ± 0.04	0.15 ± 0.06	< 0.001
TISA (mm^2^)			
TISA 250	0.02 ± 0.01	0.03 ± 0.01	< 0.001
TISA 500	0.04 ± 0.02	0.08 ± 0.03	< 0.001
TISA 750	0.07 ± 0.04	0.14 ± 0.06	< 0.001
TIA (deg.)			
TIA 250	15.24 ± 8.14	27.81 ± 8.73	< 0.001
TIA 500	11.09 ± 5.77	21.63 ± 8.05	< 0.001
TIA 750	10.60 ± 5.48	20.74 ± 7.92	< 0.001
Lens (mm)			
Back R	5.65 ± 0.40	5.66 ± 0.41	0.729
Front R	8.48 ± 0.69	8.70 ± 0.90	0.022
Decentration	0.20 ± 0.09	0.18 ± 0.10	0.305
Tilt	5.10 ± 1.06	5.19 ± 1.42	0.537
LV	0.84 ± 0.21	0.83 ± 0.23	0.152
LT	4.89 ± 0.35	4.85 ± 0.33	0.233

*SD* standard deviation, *AC* anterior chamber, *ACD* anterior chamber depth, *ACW* anterior chamber width, *ACV* anterior chamber volume, *IT* iris thickness, *ITC* iridotrabecular contact, *AOD* angle opening distance, *ARA* angle recess area, *TISA* trabecular iris space area, *TIA* trabecular iris angle, *R* radius, *LV* lens vault, *LT* lens thickness

^†^*p*-values were calculated by paired Student's t-tests

## Results

Overall, 52 Chinese participants underwent bilateral LPIs and only one eye of each patient was randomly included in the final analysis. There were 34 men and 18 women with an average age of 62.6 ± 8.3 years. The average IOP before LPI was 16.5 ± 6.3 mm Hg. The FD-OCT measurements of the thickness of the retinal nerve fiber layer and ganglion cell complex were 106.5 ± 13.2 μm and 95.9 ± 8.2 μm, respectively. The mean values of CCT and AL in the PACS eyes were 539.65 ± 31.84 μm and 22.53 ± 0.80 mm, respectively. Both mean deviation and pattern standard deviation of these PACS subjects showed no visual defects (-1.1 ± 1.2 dB and 1.8 ± 0.7 dB, respectively).

The changes in the anterior chamber and lens parameters between pre- and post- LPI are shown in Table 1. Postoperative volume of anterior chamber was significantly higher than preoperative volume (*p* < 0.001), while ACD and ACW showed no difference after the treatment (*p* = 0.151 and *p* = 0.352, respectively). IT 750, IT 2000, and iris curvature values had decreased, indicating that iris were thinner and flatter, after LPI compared to the preoperative values (*p* = 0.006, *p* = 0.044, *p* < 0.001, respectively). Both the ITC index and ITC area of post-LPI were significantly lower than those of pre-LPI (both *p* < 0.001). The anterior angle parameters including AOD, ARA, TISA, and TIA with three radii (250 μm, 500 μm, and 750 μm) showed statistically significant increase after LPI (all *p* < 0.001). Among the six lens parameters, only front R was slightly greater than that of pre-LPI, with a statistical significance of *p* = 0.022.

ΔAOD 500 (F_11.882, 13.985_ = 6.652, *p* < 0.001), ΔARA 500 (F_4.521, 539.625_ = 14.928, *p* < 0.001), ΔTISA 500 (F_4.579, 517.686_ = 14.962, *p* < 0.001), and ΔTIA 500 (F_12.205, 11.643_ = 6.887, *p* < 0.001) showed statistically significant differences in all sectors between pre- and post-LPI. The most significant change was at 79º in the superior quadrant among all the sectors, which can be observed from the line graph (Fig. 2D–G). Post hoc analysis showed that ΔARA 500 and ΔTISA 500 at 79º were significantly greater than other sectors (all *p* < 0.05), except for 101° (*p* = 0.080 and *p* = 0.178, respectively) and 180º (*p* = 0.080 and *p* = 0.196, respectively). Moreover, we found that the inferior position of 281° exhibited statistically significant second maximum ΔAOD 500 and ΔTIA 500 among all the sectors as shown in Fig. 2.

The multivariate linear regression models were developed to evaluate the association between age, sex, ACD, IT 750, LT, and the changes in the anterior chamber angles including ΔAOD 500 (F = 3.201, *p* = 0.015), ΔTISA 500 (F = 2.604, *p* = 0.038), and ΔTIA 500 (F = 3.629, *p* = 0.008). LT showed significant correlation with ΔAOD 500 (t = 2.985, *p* = 0.005), ΔTISA 500 (t = 2.277, *p* = 0.028), and ΔTIA 500 (t = 3.164, *p* = 0.003), even after adjusting for other influencing factors. Our results showed that thicker lenses were associated with a greater increase in the anterior chamber angle after LPI (Table 2).

**Table 2:** Factors associated with the change in anterior segment parameters by multivariate linear regression

		**Regression coefficient**		
		**Unadjusted β (95% CI)**	**Adjusted**	***p***-**value**^**†**^
ΔAOD 500	Age	-0.103 (-0.234, 0.028)	-0.221	0.120
	Sex	1.215 (-1.016, 3.446)	0.147	0.278
	ICURVE	-7.847 (-28.335, 12.640)	-0.123	0.444
	IT 750	-0.929 (-22.384, 20.526)	-0.014	0.931
	LT	5.538 (2.444, 8.633)	0.502	0.001
ΔARA 500	Age	-0.070 (-0.193, 0.052)	-0.171	0.254
	Sex	1.098 (-0.992, 3.189)	0.150	0.296
	ICURVE	-2.649 (-21.849, 16.551)	-0.047	0.782
	IT 750	6.163 (-13.944, 26.270)	0.107	0.540
	LT	3.897 (0.997, 6.798)	0.400	0.010
ΔTISA 500	Age	-0.083 (-0.210, 0.043)	-0.192	0.192
	Sex	1.178 (-0.972, 3.329)	0.153	0.276
	ICURVE	-4.407 (-24.156, 15.342)	-0.074	0.655
	IT 750	4.080 (-16.602, 24.761)	0.068	0.693
	LT	4.565 (1.582, 7.548)	0.445	0.004
ΔTIA 500	Age	-0.107 (-0.236, 0.022)	-0.230	0.101
	Sex	1.092 (-1.103, 3.288)	0.132	0.322
	ICURVE	-8.753 (-28.916, 11.409)	-0.137	0.387
	IT 750	-4.311 (-25.426, 16.804)	-0.066	0.683
	LT	5.825 (2.779, 8.870)	0.527	< 0.001

*AOD* angle opening distance, *ARA* angle recess area, *TISA* trabecular iris space area, *TIA* trabecular iris angle, *ICURVE* iris curvature, *IT* iris thickness, *LT* lens thickness

^†^*p*-values were calculated by multivariate linear regression test

## Discussion

In this study, we compared the parameters of the anterior segment before and after LPI, using CASIA 2. The change in the anterior chamber parameters between pre- and post- LPI has been evaluated by many devices, such as gonioscopy, UBM, and AS-OCT [15–17]. However, gonioscopy is a subjective examination and involves pressure on the cornea, which may lead to the distortion of the anterior chamber angle [18]. High resolution UBM is difficult to standardize due to the challenge of acquiring reproducible angle measurements before and after LPI [19]. Both gonioscopy and UBM require contact with the eye and hence pose a risk of contamination. AS-OCT allows for consistent and reproducible measurements of the angle parameters [20]. In our study, CASIA 2 was used, which can scan at a speed of 50,000 A-scans per second. Sixteen sections could be acquired within 5 s and analyzed to form a three-dimensional image of the anterior chamber [21]. The three-dimensional imaging technology of CASIA 2 measured the volume of anterior chamber directly, instead of estimation by rotating the anterior chamber angle of several cross-sectional scans for 360° [22]. Moreover, CASIA 2 measures tissue to a maximum depth of 13 mm, which is more than twice the depth recorded by the previous system. Improved penetration enhances visualization of the anterior and posterior surfaces of the crystalline lens [23].

Our results showed that the postoperative ACV and anterior angle parameters of AOD, ARA, TISA, and TIA were greater than the preoperative ones. In contrast, ITC index and ITC area were smaller after LPI than before LPI. All these results indicated that LPI was effective in opening the anterior chamber angle in the PACS. The circumferential analysis of 360º anterior chamber revealed that the superior quadrant angle increased the most after LPI, followed by the inferior quadrant angle. Multivariate linear regression model showed LT was significantly associated with the increase of anterior chamber angles.

In this study, the change of the four anterior chamber angle indices including AOD, ARA, TISA, and TIA demonstrated the effectiveness of LPI in the PACS eyes; this was supported by similar findings in previous studies [24–27]. Tun TA et al. also reported that angle opening distance area (AODA) and trabecular-iris space volume (TISV) were significantly increased after LPI when compared with their baseline by CASIA 2 [28].The decrease in both ITC index and ITC area verified the effectiveness of LPI treatment as well [29]. The absence of significant change in ACD (pre-LPI 2.03 ± 0.27 mm vs. post-LPI 2.04 ± 0.28 mm) in our study is consistent with previous studies using AS-OCT (pre-LPI 2.03 ± 0.04 mm vs. post-LPI 2.03 ± 0.05 mm) and UBM (pre-LPI 2.41 ± 0.28 mm vs. post-LPI 2.42 ± 0.30 mm) [30, 31]. Thus, our study, as well as previous studies, demonstrated that LPI led to a significant anterior chamber angle opening in a reliable and quantifiable manner, while the central ACD did not change after LPI.

Furthermore, we found a greater angle opening rate after LPI in the superior quadrant (79°) compared to that in other quadrants. A previous study reported the greatest widening of AOD was in the nasal quadrant. However, their study used absolute values to compare the change in AOD instead of ratio, which was used in our study [17]. The superior quadrant is well known to be the narrowest due to gravity [32, 33], which makes it more likely to have a significant change after LPI. Moreover, the location of laser spots in our patients was all in the superior area. The laser hole could reduce the pressure gradient between the anterior and posterior chambers, and the anterior chamber angles around it would then obtain the maximal increase in angle dimensions. Hence, LPI could open anterior chamber angle around the hole more than the other positions probably due to the constant percolation of aqueous humor through the laser spot [34]. Tracking techniques developed to study the aqueous humor outflow also proved that iridotomy hole caused a 17 times faster forward flow of aqueous humor than did ordinary thermal current, and this in turn would have increased pressure on the angle to open it wider [35].

Established ocular biometric factors associated with PACG include a smaller cornea, shallower ACD, shorter AL, increased LT, and anteriorly positioned lens [36–38]. Previous studies have found LV to be the strongest determinant of angle-closure, which explained approximately 70% of the variation in the angle width [3, 39, 40]. Measurement of ACD can detect occluded angles and has been evaluated as the screening parameter for angle-closure [41]. Both ACD and LV were associated with the lens position. Our results showed neither change in ACD nor LV after LPI, which suggested that treatment with LPI, did not alter the lens position. Similarly, previous studies carried out by AS-OCT and UBM demonstrated that there was no change in these two parameters [31, 42].

Furthermore, the mixed linear regression model analysis in our study found that thicker lens resulted in greater widening of anterior chamber angle. According to the mathematic prediction model of Tiedeman [2, 43], the increase of relative lens position (ACD + 1/2 LT) in the anterior chamber would cause greater pupillary block presented as iris contour and increase of anterior chamber angle crowding. Thus, LPI showed more significant effect in thicker lens subjects by reversing the greater pupillary block and angle crowding in these subjects. However, considering the average age of our PACS patients (62.6 ± 8.3 years old), LPI could be of limited use since lens extraction may often be needed in aged population [44]. Moreover, it's worth noting that although LPI is effective in preventing angle closure, it still needs to be prudently prescribed due to the low rate of conversion from PACS to PAC [45].

The current study had several limitations, including lacking BCVA and refractive status, a small sample size and short follow-up duration. Studies with larger sample size and longtime follow-up are required to determine the effectiveness of LPI and risk factors for angle closure in PACS after LPI. Furthermore, although the circumferential analysis of anterior chamber of CASIA 2 is a great advancement over the previous AS-OCT, it is still based on 16 images from different clockwise degrees instead of complete imaging of the anterior chamber; this could potentially miss information and cause errors. Besides, current CASIA 2 is unable to visualize the anatomical structures behind the iris. Lastly, evolvement of other parameters related to the ciliary body would also be helpful to reveal the mechanism and effectiveness of LPI in more detail.

## Conclusions

In our study of 52 patients, we found that LPI is effective in angle opening in PACS. We also revealed using CASIA 2 that the anterior angle was widened without change in position of the lens after LPI in PACS eyes. The anterior chamber angle in the superior quadrant around the laser hole showed the maximum widening. Eyes with higher LT are likely to have a greater angle opening effect after LPI.

## Data Availability

The data sets used and/or analyzed during the current study are available from the corresponding author on reasonable request.

## Abbreviations

AC: Anterior chamber
ACD: Anterior chamber depth
ACV: Anterior chamber volume
ACW: Anterior chamber width
AL: Axial length
AOD: Angle opening depth
ARA: Angle recess area
AS-OCT: Anterior-segment optical coherence tomography
CCT: Central corneal thickness
FD-OCT: Frequency-domain optical coherence tomography
ICURVE: Iris Curvature
IOP: Intraocular pressure
ITC: Iridotrabecular contact
LPI: Laser peripheral iridotomy
LV: Lens vault
PACS: Primary angle-closure suspect
PACG: Primary angle-closure glaucoma
POAG: Primary open-angle glaucoma
TIA: Trabecular iris angle
TISA: Trabecular iris space area
UBM: Ultrasound biomicroscopy
